# Lupus-Mediated Kidney Damage: Lupus Nephritis or Collapsing Glomerulopathy?

**DOI:** 10.7759/cureus.14468

**Published:** 2021-04-13

**Authors:** Angel De La Cruz, Haider Ghazanfar, Nayrobi Peña, Rabih Nasr

**Affiliations:** 1 Internal Medicine, BronxCare Health System, Bronx, USA; 2 Nephrology, BronxCare Health System, Bronx, USA

**Keywords:** systemic lupus erythematosus, autoimmune disease, lupus nephritis, collapsing glomerulopathy, end stage renal disease

## Abstract

Systemic lupus erythematosus (SLE) is a systemic autoimmune disease. It has a myriad of presentations and can involve almost every organ. Its effects on the kidney hold critical importance because patients can ultimately progress to end-stage renal disease (ESRD) if inadequately treated. There are many published cases of collapsing glomerulopathy (CG) in patients with SLE. However, there are not many cases reported of both SLE-related CG and lupus nephritis. Based on our review of the medical literature, fewer than 25 cases have been written about this finding. There are no guidelines to manage these types of patients. Lupus causing CG poses particular challenges in terms of management, particularly treatment options. We present a case of a 47-year-old female who was found to have biopsy findings of both CG and lupus nephritis.

## Introduction

[This abstract of this article was previously presented as a meeting abstract at the New York American College of Physician Conference in February 2020; Mount Sinai Graduate Medical Education Research Day in June 2019; and Society of Hospital Medicine, New York City/Westchester Chapter Meeting in November 2018.]

Systemic lupus erythematosus (SLE) is a systemic autoimmune disease. The exact prevalence of SLE in the United States is not known. Depending on the region, it varies from 20 to 150 cases per 100,000 [1-3]. According to a study, the prevalence of SLE was higher in black persons than white persons [4]. In the same study, it was shown that the prevalence of SLE was ten-fold higher in females as compared to males [4]. The majority of SLE patients have disease onset between the ages of 16 and 55.

SLE has multifactorial etiologies, which include hormonal, immunologic, genetic, and environmental factors. It can present with a myriad of presentations and can involve almost every organ. Its effects on the kidneys hold critical importance because nephritis and infection are the leading cause of death in the first decade of disease. Black and Hispanic populations tend to have more severe renal involvement than the white population [5].

Lupus nephritis is a well-known entity that can lead to the development of end-stage renal disease (ESRD) if inadequately treated. Lupus causing collapsing glomerulopathy (CG) presenting as a nephrotic syndrome is less common and poses particular challenges in management and treatment options. We present a case of a 47-year-old female who was found to have CG due to lupus nephritis.

## Case presentation

Our patient is a 47-year-old female who presented to the ED because of worsening bilateral lower extremity swelling, mild facial swelling, and epigastric discomfort for the past two months. Her past medical history was significant for hypertension, diabetes mellitus, and hyperlipidemia. She has been on chronic proton pump inhibitor (PPI) therapy. She denied non-steroidal anti-inflammatory drugs (NSAID) intake or herbal use. She denied smoking, alcohol, or illicit substance use. She was told that she had a kidney problem and was started on furosemide in her home country.

**Figure 1:**
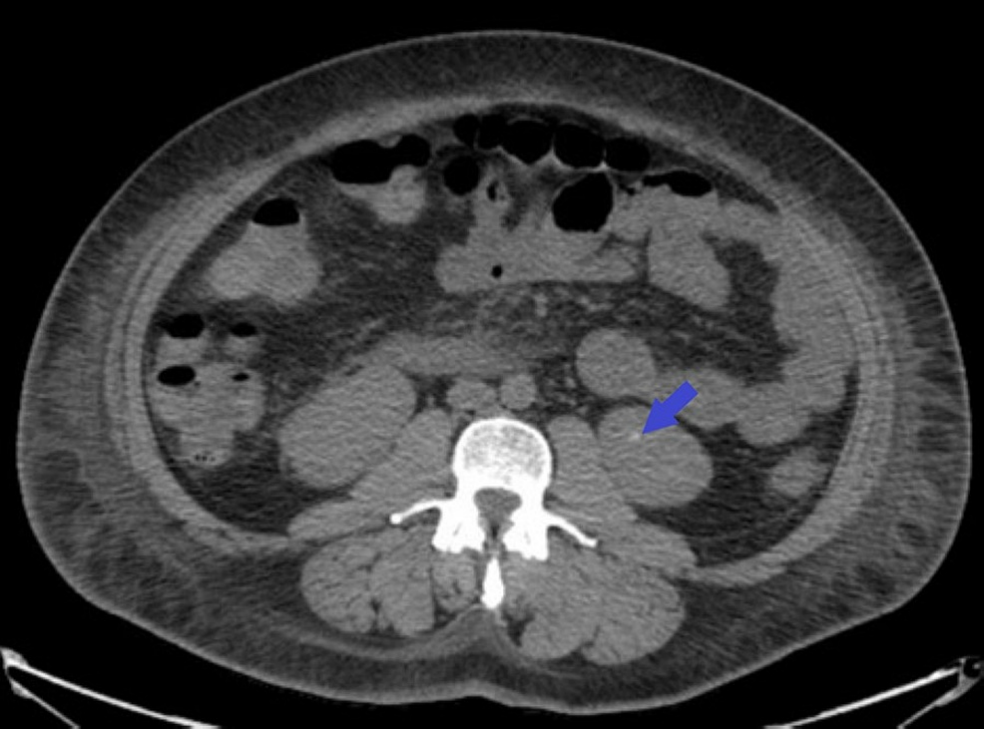
CT abdomen showing punctate left renal lower pole calcification (blue arrow).

At the time of admission, she was found to have a creatinine of 6.3 mg/dL, blood urea nitrogen (BUN) of 68 mg/dL, glomerular filtration rate (GFR) of 7.58 mL/min/1.73 m2, and potassium of 6.0 mEq/L. Repeat urine analysis showed proteinuria. Urine protein was 2929 mg/dL, and urine creatinine was 149 mg/dL. Urine albumin was >400 mg/dL. C3 and C4 complements were found to be low. ANA Screen (1:40) was positive, and it showed a homogenous pattern. Anti-double stranded DNA antibodies, myeloperoxidase, proteinase 3 AB, kappa lambda chain ratio, Sjogren’s antibody, rapid plasma reagin (RPR) titer, anti-RNP, and anti-SM were negative. The serum immunofixation study was negative. Abdominal ultrasound revealed echogenic kidneys consistent with medical renal disease and no evidence of obstruction. Ultrasound extremity veins showed no evidence of deep vein thrombophlebitis in the lower extremities. CT abdomen showed punctate left renal lower pole calcification. This has been shown in Figure 1.

**Figure 2:**
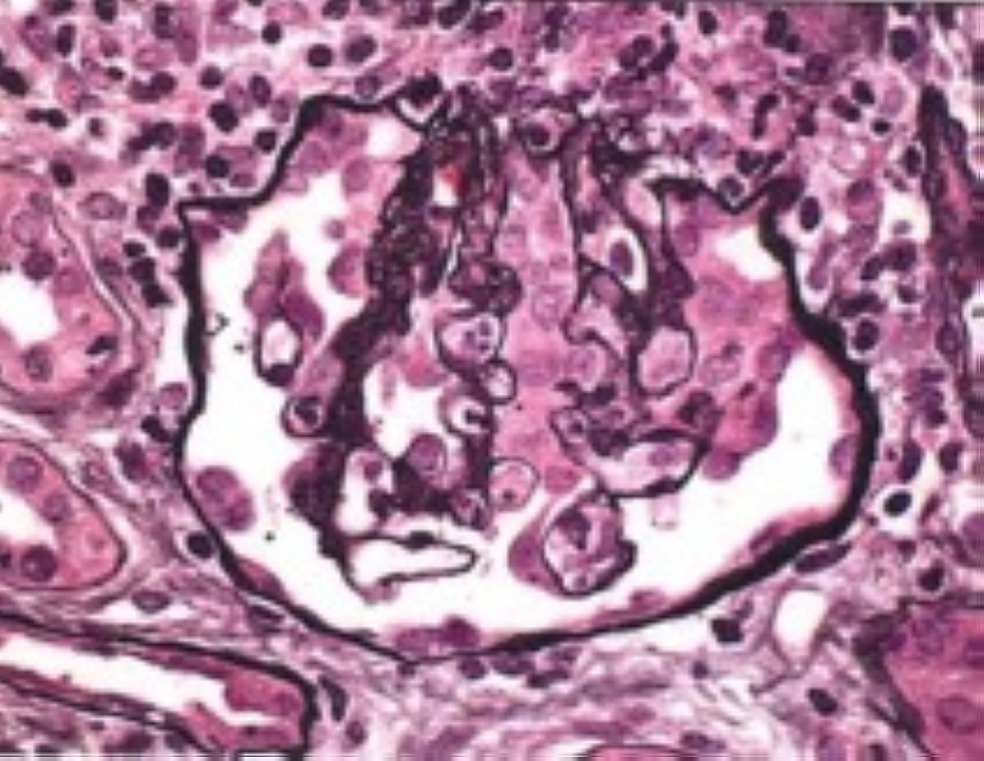
Light microscopy showing podocyte hyperplasia (x400).

**Figure 3:**
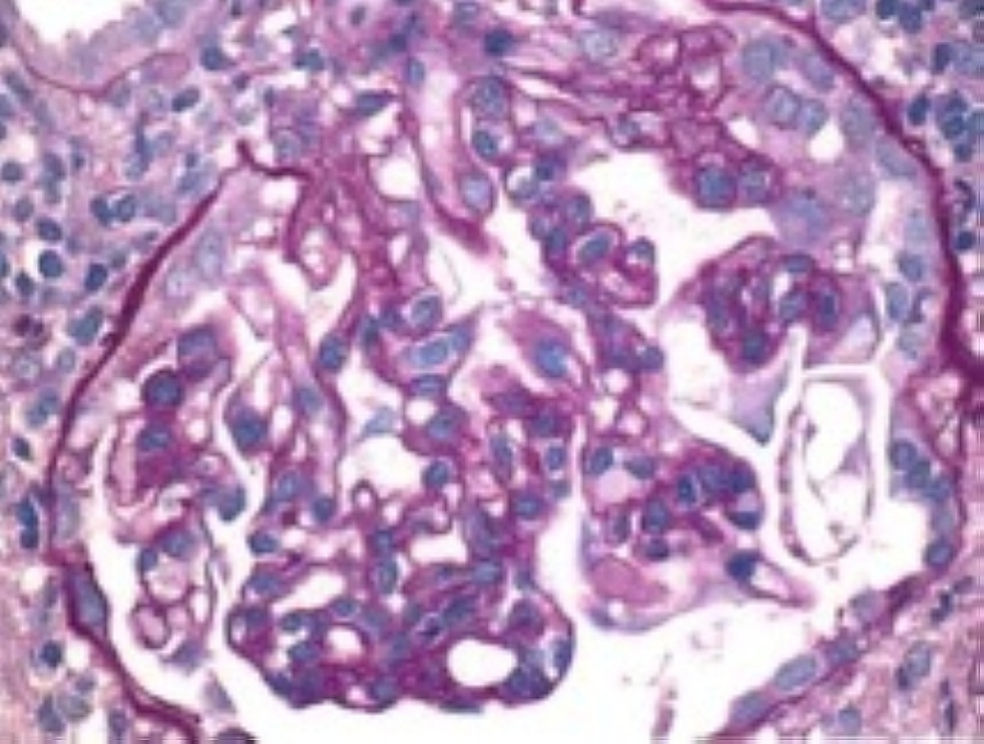
Light microscopy showing collapsing sclerosis (x400).

**Figure 4:**
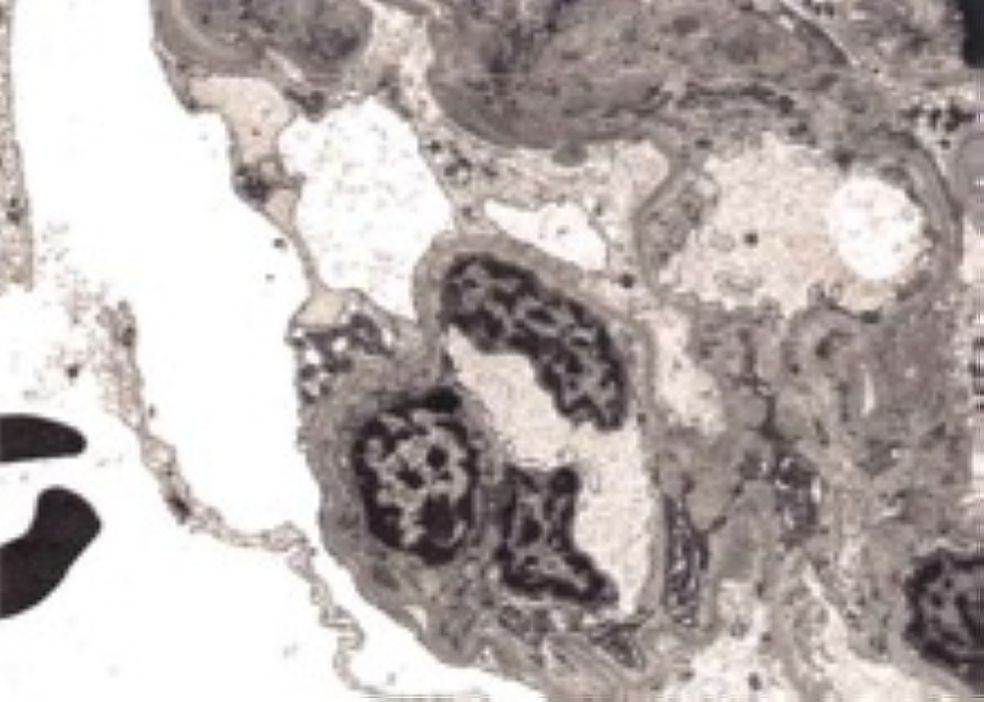
Electron microscopy showing diffuse effacement.

**Figure 5:**
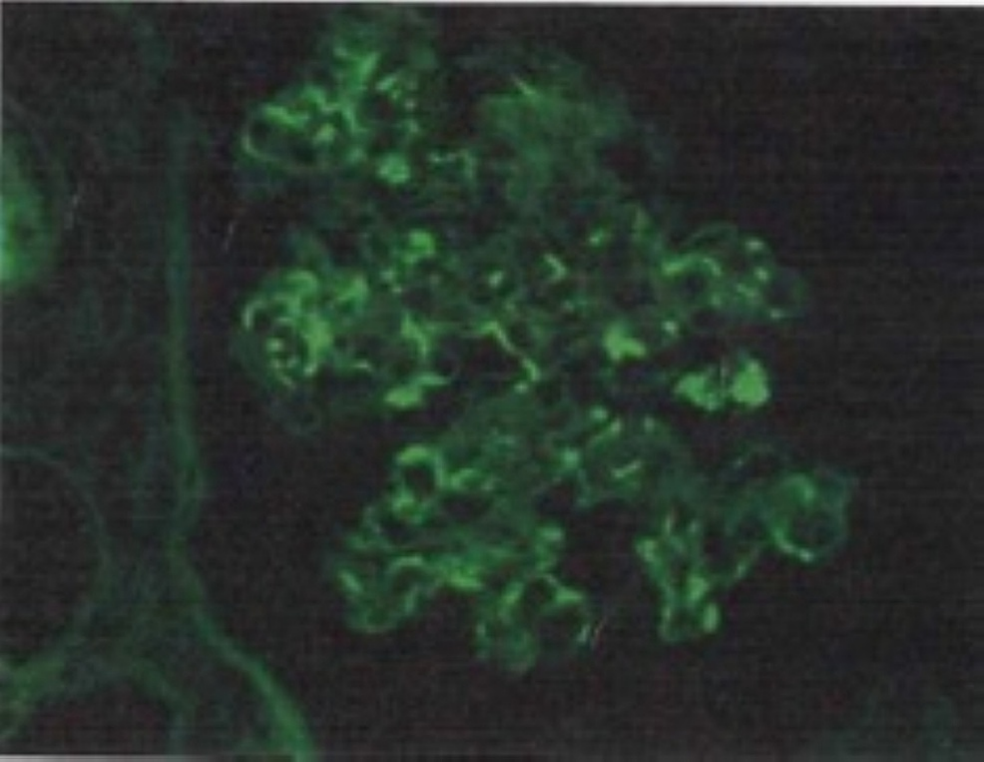
Immunofluorescence microscopy showing segmental mesangial and capillary wall immunoglobulin G deposits (x400).

She underwent a renal biopsy, which showed CG superimposed on focal glomerulonephritis, immune complex type suggestive of lupus podocytopathy (collapsing variant) superimposed on lupus nephritis class III. It also showed tubular atrophy and interstitial fibrosis. Immunofluorescence shows mesangial and segmental peripheral capillary wall staining. Immune type electron-dense deposits were seen most extensively in the mesangium with few segmental subendothelial and tubular basement membrane deposits and endothelial tubuloreticular inclusions. Overall findings were more typical of lupus podocytopathy (collapsing variant) superimposed on lupus nephritis class III. This has been shown in Figures 2, 3, 4, and 5.

She was started on IV pulse steroid treatment for three days and one dose of cyclophosphamide. She was advised to be admitted for closer observation during pulse steroid therapy due to severe renal impairment. She was discharged on oral prednisone 1mg/kg/day and monthly Cytoxan IV infusions. Prednisone was tapered down, decreasing 5 mg every two weeks. She was advised to complete nine months of Cytoxan therapy, after which the patient will be switched to Azathioprine. She tolerated the therapy and had no complications. Her creatinine improved from 6.3 mg/dL to 2.8mg/dL. She went back to her home country after being treated for about one month.

## Discussion

The only clinical finding in lupus nephritis can be proteinuria with normal or elevated serum creatinine [6]. This necessitates the use of annual urine analysis. That is why it is essential to do annual urine analysis in patients with SLE. According to a review article, proteinuria was reported in 100% of patients with lupus nephritis [7]. In the presence of hypoalbuminemia, proteinuria is usually seen in a patient with lupus nephritis having a nephrotic syndrome component. The presence of hematuria, leukocyturia or casts in the urine, low complement (C3 and C4) levels, and elevated anti-DNA titers are often associated with active lupus flare.

A kidney biopsy is essential in diagnosing the class of lupus nephritis. Lupus nephritis is divided into six different classes based on the findings of renal biopsy. Various genetic factors can influence the clinical picture of renal disease in SLE [8]. According to some studies, polymorphisms in the immunoglobulin receptor alleles Fc gamma Rlla-H131 have been linked to increased susceptibility in the development of lupus nephritis in SLE patients [9]. Each class has distinct clinical and prognostic characteristics. In about 50-75% of patients, their lupus nephritis class might transform into another class of lupus nephritis [10-12].

Lupus nephritis is a very well-known entity with clearly established treatment options. Nevertheless, at this time, there are no guidelines to manage patients with both SLE-related CG along with lupus nephritis. The treatment options and duration of treatment for lupus nephritis depend on the lupus nephritis class [13]. For the most part, the medications used in lupus nephritis are glucocorticoids, mycophenolate, cyclophosphamide, and azathioprine.

According to the American College of Rheumatology guidelines for the management of lupus nephritis [7], in patients with class III/IV disease, induction therapy should consist of mycophenolate for six months or cyclophosphamide, plus glucocorticoids. Mycophenolate is preferred in Hispanics and African-Americans. Regarding the cyclophosphamide approaches, two approaches can be taken: low-dose cyclophosphamide and high-dose cyclophosphamide. In the low-dose approach, after using cyclophosphamide, therapy is followed by maintenance with mycophenolate or azathioprine. In the high-dose approach, only cyclophosphamide is used. After the induction therapy is completed, an assessment is done to see whether the patient improved; and further management is planned accordingly.

The patient described in this case report was started on steroids and cyclophosphamide, with significant improvement in the kidney function tests. Serum creatinine levels improved from 6.3 mg/dL to 2.8 mg/dL after being treated for one month. In a case report by Fredrick et al. [14], a 30-year-old female with lupus nephritis was treated with cyclophosphamide (monthly injections) and prednisolone induction therapy for lupus nephritis. In this case, the patient’s creatinine normalized after two months of treatment. In another case report, a 36-year-old female with lupus nephritis was treated with steroids and mycophenolate, and an improvement in creatinine levels from 5.2 mg/dL to 0.7 mg/dL was seen in four weeks [15]. Contrasting, there have been cases described of patients that have never achieved normal creatinine levels after being treated for months. Our patient had a significant improvement in creatinine level.

Other than the conventional drugs used to treat lupus' renal effects, other novel therapies are under study. Although it is not considered standard therapy, belimumab has been studied in patients with lupus nephritis. As described by Margiotta et al. [16], belimumab can be especially useful in patients with intolerance to mycophenolate. Its use has also been reported in patients with lupus nephritis refractory to cyclophosphamide therapy.

In regards to the management of focal segmental glomerulosclerosis, research has demonstrated no difference in the overall response to the management with glucocorticoids among all the histologic variants of focal segmental glomerulosclerosis [17]. What is not clear is whether or not having the combination of both, CG and lupus nephritis, improves steroids' response. Further studies are required to learn more about this condition, and at this time, it poses unique challenges, a reason why management has to be individualized.

## Conclusions

There are no established guidelines to manage patients with the biopsy findings of the patient described. So, at this time, treatment has to be individualized. We suggest further studies to focus on the matter in order to develop guidelines to effectively manage these patients.
